# Successful Aponeurotomy in Springhalt

**Published:** 1892-03

**Authors:** S. J. J. Harger

**Affiliations:** Professor of Anatomy and Zoötechnics in the Veterinary Department of the University of Pennsylvania


### SUCCESSFUL APONEUROTOMY IN SPRINGHALT.

S. J. J. Harger, V. M. D.

*Professor of Anatomy and Zoötechnics in the Veterinary Department of the University of Pennsylvania.*

The theory of the elasticity of the tibial aponeurosis as a cause of springhalt appears to have been but little regarded by the American veterinarian, and cases of its section, successful or unsuccessful, have, to my knowledge, not been reported in veterinary literature. The thickened triangular fasiculus of the tibial aponeurosis in front of the hock, recognizable to the fingers on the exterior, extending from the anterior surface of this region to the tendon of the anterior extensor of the phalanges at the middle of the cannon, can, if it be very elastic, produce a sudden and spasmodic flexion of the cannon by its recoiling during flexion, having been stretched during extension of the metatarsus.

Such a condition exists in the camel and the elephant, in which both the antibrachial and the tibial aponeuroses are very thick, elastic, prolonged to the foot, and act like India-rubber springs in flexion of the foot.

Last June my attention was called to quite a well-bred horse, about six years of age, which had been affected with the so-called springhalt in the off hind member, at the time of his purchase two months previous. The spasmodic "jerk" was quite decided and not intermittent! No further history was obtainable.

Tibial aponeurotomy was the only advisable treatment. The horse having been cast on the near side, and the off limb drawn forward toward the neck with the side-line above the hock, a very small cutaneous incision was made over the tendon of the latteral extensor of the phalanges, about two inches below the upper extremity of the cannon ; a long, curved, probe-pointed tenetome being inserted, was pushed toward the internal side, passing between the skin and the aponeurosis (cannon flexed), and the latter was cut, usually with a snap, by drawing the instrument outwardly and toward the bone (cannon extended) ; the tenetome, without being removed, passed under the tendon of the lateral flexor from without to within and cut the latter transveresly. There is no danger of cutting the tendon of the anterior extensor of the phalanges, it being separated from the aponeurosis by a space the width of a finger. The wound healed kindly. The animal was kept in the stable until the inflammatory action had ceased ; the altered movements had rapidly disappeared, and at the end of six weeks the animal was practically cured.

I have never witnessed the decided knuckling forward of the fetlock, immediately after the operation, as mentioned by some authors.

The results of the operation performed by me in a number of other cases were negative. It seems to be especially contra-indicated when the posterior crural and tibial muscles are at fault, manifested by an excessive elevation of the point of the hock rather than flexion of the cannon.

Springhalt being probably due to a variety of causes, this operation is applicable only to a limited number of instances.

